# Advocating for Paid Maternity Leave and Workplace Lactation Policy Reform and Implementation: Lessons Learned From Indonesia, Nigeria, the Philippines and Vietnam

**DOI:** 10.1111/mcn.13784

**Published:** 2024-12-11

**Authors:** Michelle E. Anderson, Justine McGowan, Jessica Escobar‐DeMarco, Sujata Bose, Edward A. Frongillo, Laura Ferguson

**Affiliations:** ^1^ University of Southern California Institute on Inequalities in Global Health Los Angeles California USA; ^2^ Department of Public Health Sciences University of North Carolina at Charlotte Charlotte North Carolina USA; ^3^ ELEVATE Nutrition (Monitoring, Evaluation, Research, and Learning) Washington DC USA; ^4^ Department of Health Promotion, Education, and Behavior University of South Carolina Columbia South Carolina USA

**Keywords:** advocacy, breastfeeding, employment, maternity leave, policy change, social protection, workplace lactation

## Abstract

Maternity protection policies are designed to preserve the health of working women and their infants, support optimal infant and young child nutrition through breastfeeding and prevent workplace discrimination against women. The aim of this study was to identify how advocates may be able to effectively advance maternal leave and workplace lactation policies, two key maternity protection policies, and does so through an exploration of advocacy efforts in Indonesia, Nigeria, the Philippines and Vietnam. A desk review of programme and policy documents and 20 key informant interviews with diverse stakeholders explored advocacy efforts in each of the four countries. Thematic analyses of documents and interviews identified key considerations, challenges and success factors within each country context. These lessons can inform maternity protection policy reform efforts more broadly. Study findings show that effective, context‐specific advocacy rests on strong partnerships with traditional and nontraditional stakeholders informed by opinion leader research and strengthened through various avenues of consensus‐building. Contextual considerations are essential for identifying attainable policy asks and developing an advocacy strategy, with attention to a country's governance structure and availability of funding for social protections. Lastly, advocacy efforts may be most successful by presenting expanded paid maternity leave and breastfeeding‐friendly workplaces as parts of a set of social protections with synergistic benefits. These lessons are intended to help inform how policy advocates can better design and implement advocacy approaches for maternity protection and entitlement policies.

## Summary

- Successful advocacy for paid maternity leave and workplace lactation policies calls for evidence generation that identifies specific policy reform needs, the extent of interest from policymakers and the public, a policy window and effective angles for approaching champions and stakeholders.
- Stakeholder engagement requires mapping stakeholders, their influence and interests, how to best appeal to them and who can help convene appropriate stakeholders for consensus‐building.
- Realistic policy windows and likely champions are strongly influenced by each country's governance structure and ability to fund paid maternity leave.
- Supporting local actors in implementing paid maternity leave and workplace lactation policies can promote their effectiveness and sustainability.
- Advocacy efforts may have greatest impact by presenting expanded paid maternity leave and workplace lactation policies, along with other maternity protection policies, as a set of social protections with synergistic benefits.

## Graphical abstract

Effective advocacy for paid maternal leave and workplace lactation policies requires strong stakeholder partnerships and joint evidence generation and consensus‐building. Governance structures and social protection funding are key considerations for identifying attainable policy goals. Advocacy should present paid maternal leave and workplace lactation policies as social protections with synergistic benefits.

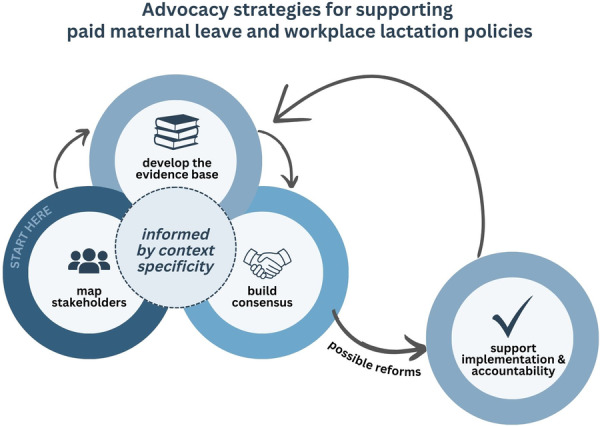

## 1 Introduction

Maternity protection policies are designed to prevent harm to the health of women^1^ who are pregnant, who recently gave birth and/or who are breastfeeding; to prevent harm to their infants' health by promoting optimal nutrition practices; and to protect them from job discrimination due to pregnancy, taking maternity leave or taking time to breastfeed while working (International Labour Organization [ILO] n.d.). These policies are necessary to provide mothers with space and time to recover from childbirth and breastfeed their babies (World Health Organization and UNICEF 2019).

Global public health guidance states that infants should ‘initiate breastfeeding within the first hour of birth and be exclusively breastfed for the first 6 months of life—meaning no other foods or liquids are provided, including water’ (World Health Organization [WHO] n.d.a; World Health Organization and UNICEF 2003, 2021). Feeding infants only breast milk from birth to 6 months of age can guarantee a safe, clean, nutritious food source with antibodies that help protect against illnesses. After 6 months, children should continue to breastfeed for up to 2 years or beyond, while also beginning to eat safe and nutritious complementary foods. When recommended breastfeeding practices are not met, children experience increased vulnerability to disease, resulting in increased rates of morbidity and mortality (Ahsan, Jain, and Walters 2022). Among health benefits such as healthy brain development and growth, breastfed children are less prone to diabetes and less likely to be overweight later in life (Pérez‐Escamilla et al. 2023). The health benefits also extend to mothers; women who breastfeed have a reduced risk of breast and ovarian cancers (WHO n.d.a; World Health Organization and UNICEF 2003, 2021; Ahsan, Jain, and Walters 2022). Maternity protection policies support exclusive and continued breastfeeding and, thus, healthy infants and mothers.

The legal and policy framework relevant to breastfeeding and child nutrition goes far beyond workplace policies, including efforts to protect against the harmful marketing of breast milk substitutes (WHO 1981), which have been studied elsewhere. Returning to work is one of the greatest challenges for exclusive breastfeeding, but particularly in workplaces that are not breastfeeding‐friendly (ILO n.d.). International standards for providing maternity protections to female workers are meant to guide national legislatures in drafting policies that will help initiate, establish and maintain recommended breastfeeding practices (WHO n.d.b).

Despite international standards and, in some cases, corresponding national laws, maternity protections often fall short. In many cases, there are few or no relevant laws in place. Existing laws may not cover all sectors or be fully implemented or effectively enforced. Globally, many women lack access to paid leave before and after childbirth, both in the formal and informal economies. Others experience working conditions that limit their ability to breastfeed, jeopardizing women's ability to both participate in the workforce and provide recommended nutrition to their newborns (ILO 2000a, 2000b). Significant legal and policy reforms are needed to support families in following WHO recommendations for exclusive and continued breastfeeding to achieve optimal nutrition.

The importance of maternity protection policies in supporting exclusive breastfeeding and thus improving health outcomes is well recognized (Kim, Hwang, and Kim 2021; Amanda Hickey, Henning, and Sirois 2022). A recent study examined breastfeeding determinants at several socioecological levels and found that health systems, workplaces, communities and households were the main settings that influence breastfeeding and that breastfeeding interventions need to be multisectoral and supported by sound health and social policies (Pérez‐Escamilla et al. 2023). Recent studies in Vietnam and South Korea have examined female workers' perceptions of maternity protection policies and how implementation gaps limit their effectiveness (Nguyen et al. 2022; Kim, Hwang, and Kim 2021). There is a significant body of evidence demonstrating the cost‐effectiveness of expanding maternity protections (Siregar et al. 2019; Ulep et al. 2020) and the costs of not breastfeeding, such as higher rates of maternal and child mortality and economic losses (Walters et al. 2016; Walters, Phan, and Mathisen 2019; Ahsan, Jain, and Walters 2022). While several articles identify limitations in existing maternity protections and the need for reform, many are rooted in a single‐country case study and provide few recommendations for contextually adaptable advocacy. An exception is the ‘Becoming Breastfeeding Friendly’ initiative, which provides a toolkit outlining how a country can score its breastfeeding environment, identify gaps and propose, scale and track policy reform (Hromi‐Fiedler et al. 2019; Buccini et al. 2019; Pérez‐Escamilla et al. 2018). Other literature evaluating advocacy strategies for maternity protection reform focuses primarily on the United States (Raabe and Theall 2016; Steinman et al. 2017).

Alive and Thrive (A&T), managed by FHI 360, is a global nutrition initiative to save lives, prevent illness and ensure healthy growth and development through improved breastfeeding, complementary feeding and maternal nutrition practices (Alive and Thrive 2017). This study synthesized and distilled learnings from A&T's maternity protections advocacy in Indonesia, Nigeria, the Philippines and Vietnam by examining advocacy efforts for paid maternity leave and breastfeeding‐friendly workplace policy changes, two key maternity protection policies. The aim of the study was to identify how advocates, considering context, can effectively champion these policies and their implementation.

## 2 Methods

To identify how, across four countries, A&T advocated for policy changes related to paid maternity leave and workplace lactation policies and what strategies and activities were most effective, this study explores the following research questions:

- How did A&T use research and technical assistance to advance policy advocacy and strengthen paid maternity leave and workplace lactation policies in the public, private and informal sectors?
- How were different stakeholders involved in policy reform efforts?
- What strategies did A&T adopt to support better implementation of paid maternity leave and workplace lactation policies?

### 2.1 Study Setting

Indonesia, Nigeria, the Philippines and Vietnam were selected to represent the geographies where A&T has been involved in advocacy around paid maternity leave and workplace lactation policies. The selection of study countries was also shaped by the availability of programme documents and publications for the desk review, and recommendations from A&T staff. At the time of interviews, A&T's maternity protections‐related work was ongoing in all of the study countries, either via continued advocacy efforts by a country team (as in Nigeria and Vietnam) or through strategic technical assistance to governments via A&T's regional office (as in Indonesia and the Philippines).

Table 1 provides basic contextual information for each study country.

**Table 1:** Characteristics of study countries.

Country	Geographic subregion	World bank classification based on gross national income	Approximate population	Governance structure	Centralized versus decentralized government
Nigeria	West Africa	Lower‐middle‐income (LMIC)	230 million	Federal Presidential Republic	Decentralized: States have considerable autonomy, with a federal system dividing powers between national and regional governments.
Indonesia	Southeast Asia	Lower‐middle‐income (LMIC)	278 million	Unitary Presidential Constitutional Republic	Decentralized: Significant autonomy granted to provinces, especially after reforms in the late 1990s.
The Philippines	Southeast Asia	Lower‐middle‐income (LMIC)	117 million	Unitary Presidential Constitutional Republic	Decentralized: Local government units have significant autonomy; power devolved to provinces, cities and municipalities.
Vietnam	Southeast Asia	Lower‐middle‐income (LMIC)	100 million	Socialist Republic (Communist one‐party state)	Centralized: Power is concentrated within the Communist Party; provinces have limited autonomy.

Table 2 provides an overview of the international standards for paid maternity leave and workplace lactation entitlements set by the ILO Maternity Protection Convention, 2000 (No. 183), and Maternity Protection Recommendation, 2000 (No. 191), compared to the laws and policies (at the time of writing) in Indonesia, Nigeria, The Philippines and Vietnam.

**Table 2:** ILO standards and current country policies on paid maternity leave and workplace lactation.

ILO standard	Indonesia	Nigeria	The Philippines	Vietnam
Maternity leave duration (not less than 14 weeks, recommended 18 weeks).	3 months, extendable in cases of complications.	Public sector: 12 weeks (longer in certain states). No guarantees for private or informal sector employees.	105 days (approx. 15 weeks), extendable in cases of complications.	6 months (26 weeks).
At least two‐thirds of previous earnings, with full salary recommended where possible, paid through social insurance or public funds.	100% salary for the first 3 months, paid by employer.	Public sector: 100% salary, paid by government. Private sector: Employer is responsible for any voluntary maternity leave pay.	100% salary, paid jointly by the social security scheme and employer.	100% salary, paid from social insurance.
One or more daily breaks or reduced hours to breastfeed.	1 h/day	Public: 2 h/day Private: No legal requirement for breastfeeding breaks.	At least 40 min/day	At least 60 min/day
Breastfeeding facilities near/at the workplace.	Not mandatory but encouraged.	Not required.	Required.	Required for companies with over 1000 female employees.

*Note:* Data for ILO are from (2000a, 2000b). Data for Indonesia are from Heyder et al. (2024). Data for Nigeria are from Alive and Thrive (2019, n.d.b). Data for the Philippines are from Republic of the Philippines (2010), Santos and Tan (2020) and Alive and Thrive (2021b). Data for Vietnam are from the Vietnam National Assembly (2014, 2019).

### 2.2 Study Design and Methods

The two primary data collection methodologies for this qualitative research were a desk review and in‐depth interviews (IDIs).

#### 2.2.1 Desk Review

A desk review of A&T documents from the four study countries included project‐, country‐ and regional‐level activity reports; technical reports; meeting reports; tools and guides; advocacy materials; media articles; and peer‐reviewed articles, including economic impact studies. Additional sources not produced and provided by A&T, including global and country‐specific resources on paid maternity leave and workplace lactation policies, were reviewed to provide solid normative and theoretical grounding. A data extraction matrix was created in Microsoft Excel and populated following the themes of the study objectives. These included, for example, information on the activities undertaken, challenges and successes, political dynamics, other stakeholders involved and outstanding questions for possible inclusion in the qualitative interviews. The desk review was an iterative process in which one member of the study team extracted the data and two other members of the study team reviewed the extraction matrix for consistency, clarity and comprehensiveness; the matrix was amended as needed.

#### 2.2.2 Qualitative Data Collection and Analysis

The qualitative data collection involved IDIs conducted virtually with staff from the A&T home office in the United States, A&T regional‐ and country‐level offices, A&T partner civil society organizations (CSO) and external stakeholders (such as government staff) in the study countries. One general interview guide was developed based on the research questions and desk review findings and adapted for each national context based on country‐specific desk review findings, and, when appropriate, a participant's role in the advocacy efforts. The IDI guides are available from the corresponding author on request.

Sampling was purposive to include different types of stakeholders and maximize learning from the most appropriate participants. A&T provided lists of A&T staff as potential interview participants based on their involvement in relevant advocacy activities and their ability to be interviewed in English. Selected individuals were invited to participate voluntarily, but their identities were not shared with A&T home office staff. Two members of the study team were present for each interview; these members were independent from A&T. Interviews were audio‐recorded and transcribed by Zoom. Members of the research team edited the transcriptions for accuracy.

The number of interview participants per country depended on participants' level of information and the availability and responsiveness of potential interviewees. Twenty‐four people were contacted; five declined to participate, and two did not respond. Twenty interviews were conducted (see Table 3) with 17 people. One participant was interviewed twice, and another was interviewed three times because of their expertise across multiple countries. In cases where a participant was interviewed more than once, each interview focused on a different country. Despite the country focus of each interview, in some cases, participants with knowledge of multiple countries gave examples from a country that was not intended to be discussed during that session.

**Table 3:** IDIs conducted.

Focus country	Interviews	Institutions
Vietnam	4	A&T staff, local partner CSO
Philippines	4	A&T staff, local partner CSO
Nigeria	5	A&T staff, local partner CSO, private sector network, government advisor
Indonesia	4	A&T staff, university research partner
Home office (HO)	3	A&T staff
Total	20	

These 20 interviews were anonymized and assigned a code based on the focus country and interview sequence.

Thematic analysis was carried out by two members of the study team not associated with A&T through an iterative process, and reviewed by a third member, also not associated with A&T. Interview data were analysed alongside data collected in the desk review. Data across sources jointly fed into answering all research questions as relevant. Researchers triangulated facts and emerging themes across different data types and through follow‐up with interview participants.

#### 2.2.3 Ethical Approvals

The study protocol was presented to FHI 360's Office of Institutional Research Ethics and received a non‐research determination. It was also submitted to the Kaduna State Government in Nigeria and was exempt from research ethical approval. IRB approval was not required for Vietnam, the Philippines or Indonesia due to the study's non‐research determination. Nevertheless, the principles of informed consent, anonymity and confidentiality were applied during the interview processes.

## 3 Results

This results section presents the advocacy strategies described by participants across country teams, which are further elaborated on or contextualized using data from the desk review of A&T documents. Results are organized not by research question but instead trace advocacy strategies in a stepwise manner that reflects interview participants' descriptions of emergent themes. These include stakeholder mapping; evidence generation; consensus‐building; and support for implementation, monitoring and accountability. These advocacy strategies build upon each other to foster sustainable and cascading social and policy change to support paid maternity leave and workplace lactation policies and subsequently, nutrition. The end of the results section highlights participants' emphasis on the importance of context and governance structure within reform efforts.

### 3.1 Advocacy Strategy

The main activity categories relating to advocacy strategies that surfaced through the interviews included stakeholder mapping; evidence generation; consensus‐building; and support for implementation, monitoring and accountability.

#### 3.1.1 Stakeholder Mapping

A&T recognizes stakeholder mapping as critical for planning any advocacy strategy. This step identifies in‐country partners and policy champions with shared reform goals or who are otherwise influential in the relevant space, including potential opposition (Alive and Thrive n.d.a).

Recognizing that large‐scale reform requires consensus from policymakers, partner organizations and the public, stakeholder mapping should include traditional and nontraditional partners and provide a deep contextual understanding of power structures. An A&T partner in Nigeria suggested that stakeholder mapping should continuously track and respond to changes impacting policy windows or advocacy strategies, such as shifts in country priorities or government turnover (Nigeria4).

An A&T employee who works across countries stated that analysing the stakeholder landscape should always include identifying ‘ways to engage with [stakeholders] and issues that are of interest to them’ (HO2); interview participants in Nigeria affirmed that this can inform a tailored approach to stakeholder engagement (Nigeria3; Nigeria4). Participants related how the success of advocacy relies on ‘the relationships we have in‐country, what doors we can open—if we can get a seat at the table, you can get a minister to return your phone calls’ (HO1), which also requires understanding who the ‘power levers’ (Nigeria4) and ‘gatekeepers’ are to make advocacy and implementation processes smooth and sustainable (Nigeria2). While this project's interest in policy reform was rooted primarily in nutrition, there were several possible angles from which to advocate for paid maternity leave and workplace lactation policies to achieve the desired outcome.

#### 3.1.2 Developing the Evidence Base

Participants in all study countries emphasized that the strategic use of data is central to successful advocacy. A participant in Indonesia with additional familiarity of other countries' programming pointed to context‐specific evidence generation as a key strength of A&T and often the starting point for advocacy across contexts (Indonesia2). Evidence generation involves gathering data to build a case for reform, including impact data, key barriers, opportunities and levers of change and credibility of potential solutions.

##### 3.1.2.1 Identifying Policy Asks and Policy Windows

Evidence generation informs policy asks and provides a basis for contextually adapting reform efforts:

We don't just ask governments to pick a policy, cut and paste it, put their logo and then that's it. We want to make sure that they understand the need, that they understand what policy adoption and implementation will take but also more importantly […] what will the policy resolve.

Philippines2

In response to anecdotal evidence that women in Nigeria were ‘not very confident with this issue of maternity leave’ due to fear of losing employment (Nigeria2), in 2018, A&T conducted a National Maternity Entitlement Survey in partnership with UNICEF and the Ministry of Health (Alive and Thrive 2019). The survey assessed existing entitlements, women's perceptions and uptake across sectors. A participant in Nigeria noted that the survey's findings informed A&T's subsequent advocacy efforts in Nigeria, including the identification of the policy issues, specific policy asks and opportunities to act based on public support for expanding entitlements (Nigeria2).

According to participants, evidence generation also includes research to identify interest from policymakers and time advocacy efforts appropriately. Policy landscape research can help identify entry points and opportunities for consensus‐building, where to target advocacy and what points to emphasize. A participant from the Home Office provided an example of this, noting that A&T has found that while paid maternity leave and workplace lactation policy reform efforts often ground advocacy arguments in issues of employment or labour, at times, advocacy may be more effective if based on child nutrition or women's economic rights (HO2).

##### 3.1.2.2 Working With Partners and Impacted Communities to Ensure Acceptability of Reform Efforts

Acceptability, a key tenet within human rights, concerns the subjective perceptions of impacted communities regarding interventions, including policy reform efforts, and seeks to ensure that the intervention is relevant, needed and culturally appropriate for the context. This facilitates implementation and uptake. The qualitative nature of acceptability necessitates participation of impacted communities in reform efforts from their earliest stage.

A strong relationship with groups that a policy will impact is critical at all stages of the advocacy process. Connections with and insights from affected communities are vital to ensuring that policy advocates are able to identify the most relevant and impactful types of data and information, and also promotes buy‐in from these communities for the collection and dissemination of such evidence. Participants commonly described how such relationships help ensure that the evidence supports appropriate and acceptable reforms and builds consensus, further discussed below. A&T's relationship with informal workers via the Alliance of Workers in the Informal Economy/Sector (ALLWIES) in the Philippines, a collaboration advancing the visibility of informal sector workers (Alive and Thrive 2021b; ALLWIES n.d.), illustrates one approach to such engagement. Participants described how ALLWIES was consulted early on in the conceptualization of the possible reform efforts:

In our efforts for maternity leave for the informal sectors, we consulted the sector itself through ALLWIES and asked them if the study we plan to do would be helpful or complement their ongoing effort in advocating for the welfare of the female workers in the informal economy.

Philippines1

Involving the groups that a policy will serve from the earliest stages of evidence generation ‘raise[s] the issues and give[s] a voice, a real human voice, to the group, the vulnerable group in question’ (Philippines2). In the Philippines, participants reported that ALLWIES provided the call to action for policymakers, and A&T provided the evidence necessary to write and implement the policy (Philippines1; Philippines2). Their involvement also ensured the acceptability of proposed reforms, contributing to the higher likelihood of implementation and uptake of the policy; in the case of paid maternity leave and workplace lactation policies, this results in better nutrition outcomes.

##### 3.1.2.3 Considerations of Funding Sources for Maternity Entitlements

Interview data indicate that advocacy must be informed by a contextual understanding of a country's gaps in protections such as paid maternity leave and workplace lactation policies and its economic landscape, particularly a country's ability to fund these protections. For example, participants in Vietnam described that, despite interest from policymakers in expanding paid maternity leave for informal workers, there was no public funding scheme for doing so, which led them to determine that there was low feasibility of addressing the lack of paid maternity leave for informal workers without significant evidence related to costing (Vietnam1).

Similar sentiments were expressed by participants in Nigeria, where no social security scheme exists. The combination of gaps in legal and policy protections alongside gaps in opportunities to finance maternity leave informed the decision to use different advocacy approaches for different sectors in Nigeria; ‘for the public sector, we push 6‐month leave, but for the private sector, we push baby‐friendly workplaces’ (Nigeria1). Participants explained that 6‐month paid maternity leave is a more feasible reform for the public sector because the government can more easily access funds to provide paid, extended leave for its employees, whereas workers in the private sector rely on their company's willingness and ability to fund maternity leave pay (Nigeria2). Unsurprisingly, participants stated that there is limited support for 6‐month leave in the private sector (Nigeria4). Advocacy for baby‐friendly workplaces was therefore presented alongside a continued push for the expansion of paid maternity leave, with the acknowledgement that paid leave and breastfeeding‐friendly workplaces can and should work in tandem to best support breastfeeding mothers and, therefore, optimal nutrition.

Contextual evidence is necessary to address policy inadequacies with consideration for a country's financial structures. A&T effectively accounts for economic landscapes during the evidence generation stage of advocacy, which often informs an economic costing study (e.g., see Siregar et al. 2019; Ulep et al. 2020; Siregar et al. 2021) or sector‐specific policy asks.

##### 3.1.2.4 Tailoring Evidence Type and Format to Audience and Context

Participants across countries agreed that the type and format of evidence should be tailored to different audiences' concerns, motivations and mandates. Different evidence may be presented to underpin a target issue for different stakeholders; for example, while ‘a parliamentarian might focus more on the economic gain, a health minister might be interested, of course, on the health outcomes that are expected’ (Philippines2).

A&T staff from all four countries mentioned the importance of studies that highlight economic benefits as opposed to only health‐related impacts of maternity protections such as paid maternity leave and workplace lactation policies. One participant stated that:

Governments are more responsive to economic benefit. They want to know or have an idea of how much it will cost to implement to program, and it's a fair question because if it's a law, then under the law you need the state to source the funding.

Philippines2

Interview data also indicated that A&T's economic impact studies commonly filled a data gap in each country's understanding of maternity protections such as paid maternity leave and workplace lactation policies. For example, participants in Indonesia stated that before the study on the economic cost of not breastfeeding, evidence only focused on the positive health effects of breastfeeding, which did not resonate with policymakers beyond the health sector (Indonesia1; Indonesia2). A 2019 A&T study illustrated that the cost of a publicly financed, noncontributory maternity protection programme for informally employed women in the Philippines is affordable based on the economic gains from positive health and non‐health outcomes associated with improved breastfeeding rates and female labour force participation (Walters, Phan, and Mathisen 2019). A&T staff reported senators stating that ‘the A&T study provided them the missing piece for them to be able to file the bill to say that we can afford to provide maternity leave for the informal sector’ (Philippines1).

#### 3.1.3 Consensus‐Building

Building consensus with relevant champions is fundamental for advancing policy advocacy and strengthening paid maternity leave and workplace lactation entitlements in the public, private and informal sectors. Consensus‐building can take place during any and all stages of the advocacy process.

##### 3.1.3.1 Generating Support Among Policymakers and Nontraditional Stakeholders

Government was emphasized by participants as an essential stakeholder for reform efforts across the four countries. Therefore, ‘having someone [on the A&T team] who is connected and accepted by the government is essential’ (HO2). In the Philippines, A&T worked closely with the Departments of Health and Labor ‘every step of the way’ during a study on financing paid maternity leave entitlements for the informal sector (Philippines1; Indonesia1). According to one participant, this relationship allowed A&T to ‘directly communicate with the committees in […] the Senate and the House of Representatives’, where bills are filed (Philippines1). Involving government stakeholders from the earliest stages of advocacy promotes ownership and secures champions for the focus issue.

A&T staff in Vietnam and Nigeria described the process of producing evidence‐based policy papers as a method for consensus‐building with other CSOs for stronger policy asks. One participant described how, in the face of initial resistance from the Ministry of Labour‐Invalids and Social Affairs (MOLISA), the ministry responsible for Vietnam's labour code, A&T partnered with UN Women and the German Corporation for International Cooperation to demonstrate the connection between workplace lactation entitlements and gender equality in the workplace, co‐authoring a policy white paper that provided a cost–benefit analysis of workplace lactation rooms (Alive and Thrive 2020). The participant recalled that this white paper ushered support from organizations working in nutrition, labour and gender and was presented again to MOLISA, leading to the decree mandating lactation rooms in larger workplaces (Vietnam1).

Participants from multiple countries provided examples of instances in which A&T found success in partnering with nontraditional stakeholders, such as entities focused on gender equality and individual champions with access to policymakers. Examples included ‘retired government, academia’ (HO2) and, in Nigeria, the governors' wives. While the Nigeria Governors' Forum has been a key government partner in pushing for reform, a participant described how A&T ‘found out that in some of the states, it's actually the governors' wives who are the ones that are really going to help get some these policies through’, leading them to determine that the wives' interest in promoting breastfeeding for child nutrition and their influence on the governors made them strategic partners for furthering advocacy in Nigeria (HO1).

##### 3.1.3.2 Media Engagement for Consensus‐Building

Participants in multiple countries discussed efforts to engage with the media on issues of maternity protections. Participants in Vietnam described finding particular success through media advocacy, stating that media is an important space to amplify public support for an issue and to inform and increase interest among policymakers (Vietnam2). In Vietnam, A&T connected members of an online breastfeeding mothers' group to media outlets, where their personal stories about the need for workplace lactation spaces were shared alongside evidence generated by A&T on the benefits of breastfeeding‐friendly workplaces (Vietnam1; Vietnam2; Alive and Thrive 2021a). Participants shared that this strategic media engagement showed clear public support, indicating to policymakers that this reform would be welcomed (Vietnam1).

According to participants, strategic media engagement encourages action; if the public is informed about benefits behind a policy change, ‘when the policy is adopted, it will be implemented and enforced. Not only by the policymakers but also welcomed by the community’ (Vietnam1).

##### 3.1.3.3 Holding Advocacy Events for Information Dissemination and Consensus‐Building

Several interview participants highlighted the impact of holding in‐person events to reach the diverse stakeholders required for comprehensive advocacy (HO2; Nigeria2; Philippines2). A&T has facilitated consensus‐building by hosting events where strategic communication products such as policy briefs, infographics, handbooks or toolkits were disseminated. Participants described events that ranged from advocacy meetings with policymakers, consultative workshops with private sector leaders and large‐scale events. For example, on Labor Day 2021 in the Philippines, 230 informal workers and members of government convened to discuss the need for and cost of expanding paid maternity leave to the informal sector. Interview participants described the impact of this event, noting that ‘the findings and the participants' strong commitment contributed to the submission of Senate Bill 2175’ (Philippines2) on financing paid maternity leave for the informal sector, and ‘in fact, it was filed a week after our forum’ (Philippines3).

#### 3.1.4 Supporting Policy Implementation and Accountability

Legal and policy reform will only be impactful if the reforms are implemented. Activities that support policy implementation, including monitoring and accountability, should ideally be built into the policy itself. Participants across study countries acknowledged that monitoring and enforcing policy implementation were significant challenges (Indonesia1; Nigeria2; Vietnam1). Compliance may also depend on the funding mechanisms available for policy implementation; for example, a participant in Nigeria, where the government does not fund paid maternity leave for private sector employees, stated that compliance depends on a company's ability and willingness to pay the permanent employee during their leave and their temporary replacement (Nigeria2).

In cases where there is a lack of legal accountability, participants explained the ways that A&T promoted policy implementation through other means. In Nigeria, A&T participated in developing a tool for tracking nutrition indicators, including a 6‐month paid maternity leave indicator,^2^ via a scorecard maintained by the Nigeria Governors' Forum (Nigeria2). Participants in Nigeria shared the belief that the practice of governors presenting the scorecard results at a biannual forum of peers provides a helpful push for the implementation of national policies at the state level, even without the existence of legal ramifications for noncompliance (Nigeria4; Nigeria2).

A&T has also supported policy implementation via technical assistance. For example, following the Vietnamese decree mandating workplace lactation rooms for companies with over 1000 female workers, A&T developed a guideline for establishing workplace lactation rooms (Alive and Thrive 2023) and hosted consultative workshops for companies on implementation (Vietnam1). Participants said that following these workshops, two multinational companies then contracted A&T to implement workplace lactation rooms for their vendors and suppliers in Vietnam, totalling 20 factories (Vietnam1; Vietnam2). One participant in Vietnam described these activities as simultaneously providing clients with a service that ensures policy compliance and serving as an example of implementation for other companies (Vietnam2).

### 3.2 Contextual Adaptation of Advocacy Strategies

Although A&T used many of the same advocacy approaches across countries, the advocacy strategy is highly dependent on context and must include attention to how law and policy are created, enacted, implemented and enforced, who the important stakeholders are and the country's current issue priorities. While not explicit in the interview questions, contextual considerations and governance structure were highlighted by interview participants as critical factors in reform efforts.

For example, one participant highlighted how Vietnam's centralized government structure allowed for a national policy change to occur and be cascaded down to provinces; reform efforts were also successful partly due to a partner organization that was legally embedded in the National Assembly (Indonesia3, speaking on the Vietnam case). Participants in countries with decentralized governments indicated that finding a similar national‐level partner to provide access to policymakers and lend legitimacy to A&T's work would be challenging and likely ineffective (Indonesia3). Participants in Nigeria and Indonesia also indicated that while requiring greater capacity, advocacy at subnational levels is more effective for ensuring political will and follow through (Indonesia2; Nigeria2; Nigeria4). For example, participants in Nigeria described how a state‐level approach may serve as empirical evidence; ‘ultimately, if something works in one state, you want to scale it up, and then you could get a national policy developed around what was tested from a state’ (Nigeria4). This is in reference to their use of Lagos and Kaduna as ‘learning states’, where A&T piloted an advocacy process in multiple Local Government Areas before scaling up to 11 of the 36 states in Nigeria (Nigeria2).

## 4 Discussion

This study has presented findings on A&T's advocacy for paid maternity leave and workplace lactation policies in Indonesia, Nigeria, the Philippines and Vietnam. Findings include considerations, challenges and success factors within advocacy and its contextual adaptation. These findings include crucial lessons for future reform efforts for paid maternity leave and workplace lactation entitlements, and other maternity protection policies. We discuss these lessons below in the context of three areas that the findings highlight as key to advocacy: evidence, context and synergies; stakeholder involvement; and strategies to support implementation.

### 4.1 The Importance of Evidence, Context and Synergies

There is a range of existing work on the relationship between improved health outcomes and protections, such as paid maternity leave and breastfeeding‐friendly workplaces (Kim, Shin, and Donovan 2019; Steurer 2017; Albagli and Rau 2019). A&T's advocacy approach stands out in its understanding of these and other maternity protections as a set of policies‐ as opposed to each protection individually‐ that can work in concert to support the health and overall well‐being of parent and child as well as women's rights and economic empowerment (Nguyen et al. 2022; Gribble et al. 2023). As has been recommended by other research (Pereira‐Kotze, Doherty, and Faber 2022), using this fuller picture as the conceptual starting point provides opportunities to harness a more comprehensive advocacy strategy. Best practices include leveraging partnerships with nontraditional partners and finding additional advocacy entry points beyond health‐based evidence (Steinman et al. 2017), for example, through the provision of economic costing studies. Moving forward, advocates should endeavour to conceptualize and present maternity protection policies as a comprehensive package, which calls for evidence highlighting their synergistic benefits. A comprehensive package of maternity protection policies is certainly not limited to paid maternity leave and workplace lactation entitlements. It should also include guarantees for health protection, access to medical entitlements and job security during pregnancy and while breastfeeding; provisions that prohibit discrimination due to pregnancy, childbirth and breastfeeding; and policies that enable access to childcare support. While it may not be possible to implement a comprehensive package of policies immediately, understanding its benefits may allow an incremental approach towards this.

As other literature has indicated, effective advocacy requires knowledge of lawmaking processes and governance structures as well as continuous monitoring of opportunities for significant policy change based on the confluence of widespread awareness of the problem, the availability of a feasible solution and political will (also known as policy windows) (Baker et al. 2013; Kingdon 2010). Deep understanding of national and subnational governance structures and policy processes is essential for understanding entry points for advocacy. Participants across the study countries stated that this continuous monitoring should be done alongside continuous stakeholder mapping to understand and respond to shifts in country priorities, election cycles or government turnover.

### 4.2 Stakeholder Involvement

Several of this study's findings on stakeholder involvement align with existing literature, including the importance of opinion leader research and stakeholder mapping for informing nutrition policymaking processes (Weissman et al. 2020). Establishing and sustaining partnerships requires mapping stakeholders and potential opposition, their topical interests and how to best appeal to them. It is clear from this study and others that policy advocacy and reform depend on strong stakeholder networks; collaboration with other stakeholders working in the same issue areas increases capacity while building consensus with policymakers, partner organizations and the public (Michaud‐Létourneau, Gayard, and Pelletier 2019). Our findings support existing best practices by indicating the importance of having someone on each country's policy advocacy team who is connected to and accepted by the government. These findings are also relevant to other areas of health policy advocacy (David et al. 2020; Hann et al. 2015).

Our findings strengthen those of earlier research on the development of a breastfeeding‐friendly policy in Washington State, USA, which proposed, in anticipation of opposition, that the policy ask should be framed beyond only ‘individuals and health’ and the coalition developing the policy should include stakeholders beyond traditional health stakeholders (Steinman et al. 2017). The multi‐sectoral nature of maternity protections means that advocacy efforts should ensure to include stakeholders from various sectors; findings from all four study countries demonstrate that entry points for paid maternity leave and workplace lactation policy advocacy may include health, labour or gender equity, each requiring different evidence and stakeholders, both within and outside of government. In addition to indicating the far‐reaching applicability of this finding, our study builds on this concept, noting that realistic policy windows and likely champions are also informed by each country's ability to fund these policies.

### 4.3 Strategies to Support Implementation and Accountability

This study has highlighted the importance of contextually tailored advocacy, with attention to governance structures. A novel finding is that in decentralized settings, an under‐studied area for this type of work, advocacy efforts should be aimed at the subnational level alongside consultation with the national level to ensure political will and commitment to implementation. We also found that supporting local actors in implementing paid maternity leave and workplace lactation policies can promote sustainability.

Recent literature has identified a lack of monitoring systems as a barrier to implementing maternity protection policies (Maramag et al. 2023). The topics of monitoring and compliance within the context of maternity protections policy implementation would benefit from additional research in all country contexts, which might include research on the monitoring and compliance strategies that exist in countries that have successfully implemented maternity protection policies.

### 4.4 Limitations

This study used an organizational focus; by using case studies, the findings are able to speak to lessons learned from advocacy efforts by A&T. Although the findings are widely applicable outside of the context of A&T or the study countries, inclusion of other advocacy organizations would have brought additional perspectives to the lessons learned. Additionally, all interviews were conducted in English; conducting the IDIs in participants' home language may have expanded our pool of respondents and captured more nuances.

## 5 Conclusion

After over a decade of advocacy efforts, A&T has made significant contributions to paid maternity leave and workplace lactation policy reform and, therefore, optimal nutrition through support for exclusive breastfeeding in Indonesia, Nigeria, the Philippines and Vietnam. These efforts and their outcomes illustrate that successful advocacy requires understanding the specific policy reform needed, the extent of interest from policymakers and the public, a policy window and effective angles for engaging champions and stakeholders. Realistic policy windows and likely champions are informed by each country's context, including the governance structure and ability to fund maternity protections such as paid maternity leave and workplace lactation entitlements. Supporting local actors in implementing these policies can promote sustainability; however, reform efforts would benefit from more attention to supporting policy implementation and accountability. The lessons from this study can inform and direct contextually adapted efforts toward maternity protection policy advocacy and reform to advance women's and families' health and economic rights.

## Author Contributions

L.F. conceptualized this work and oversaw data analysis. L.F., M.E.A. and J.M. led drafting of data collection instruments. M.E.A. and J.M. carried out primary data collection, led data analysis and wrote the first draft of this article. J.E.‐D., S.B. and E.A.F. contributed to initial project design and provided inputs on the manuscript. M.E.A. and L.F. worked together to create the final draft. All authors approved the final manuscript.

## Conflicts of Interest

The authors declare no conflicts of interest.

## Data Availability

The data that support the findings of this study are available from the corresponding author upon reasonable request.

## Endnotes

1 Recognizing that gender‐diverse individuals, and individuals who identify as men, can be pregnant, give birth and breastfeed, this article will, however, use ‘women’ to describe the individuals protected by most maternity protection policies, as this is the terminology used in the policies of the study countries.

2 The 6‐month maternity leave indicator is based on the 2016 Abuja Breastfeeding Declaration, signed by policymakers, private sector employers and labour unions, which commits Nigeria to prioritizing key policy and programme recommendations as outlined by the 2016 Lancet Breastfeeding Series, including 6 months of paid maternity leave (Alive and Thrive n.d.b).
